# Mechanisms of Hearing Loss in a Guinea Pig Model of Superior Semicircular Canal Dehiscence

**DOI:** 10.1155/2018/1258341

**Published:** 2018-04-24

**Authors:** Bu-Sheng Tong, Zi-Yu He, Chen-Ru Ding, Juan-Mei Yang, Jing Wang, Zhao Han, Yi-Bo Huang, Na Gao, Xian-Hao Jia, Fang-Lu Chi, Dong-Dong Ren

**Affiliations:** ^1^ENT Institute and Otorhinolaryngology Department, Affiliated Eye and ENT Hospital, Fudan University, Shanghai, China; ^2^Department of Otorhinolaryngology of the First Affiliated Hospital, Anhui Medical University, Hefei, China; ^3^Shanghai Auditory Medical Center, Shanghai, China; ^4^Key Laboratory of Hearing Science, Ministry of Health, Shanghai, China

## Abstract

Defective acoustic transmission in the cochlea is closely related with various auditory and vestibular symptoms. Among them, semicircular canal dehiscence (SCD) with a defective semicircular bone is typical. Currently, the pathogenesis of SCD is usually explained by the third window hypothesis; however, this hypothesis fails to explain the variability in the symptoms and signs experienced by superior SCD (SSCD) patients. We evaluated the mechanism of hearing loss in a guinea pig model of bony dehiscence with various sizes and locations along the superior semicircular canal. Auditory brainstem responses (ABRs) and laser Doppler velocimetry were used to measure hearing loss and vibration changes before and after fenestration, as well as after restorative patching. ABR thresholds at low frequencies (e.g., 1000 Hz) increased after fenestration and decreased back to the normal range after we repaired the defect. Energy leakage from the surgically introduced third window was detected in the range of 300–1500 Hz, accompanied by increased vibration at the umbo, stapes head, and the dehiscence site, while decreased vibration was observed at the round window membrane in the same frequency range. After the patching procedure, the deviant vibrations were recovered. The degree of postfenestration energy leakage was proportional to the size of fenestration and the proximity of the fenestration site to the oval window. These results suggest that the bony fenestration of the superior semicircular canal mimics the hearing loss pattern of patients with SSCD. The decrease in perilymph wave impedance likely accounts for the auditory changes.

## 1. Introduction

Superior semicircular canal dehiscence (SSCD), initially reported by Minor et al. in 1998 [1], is a clinical entity associated with vestibular symptoms typically evoked by sound and pressure stimuli. Temporal-bone histopathological studies suggest that the superior canal of 1-2% of the population has abnormally thin overlying bone. Disruption of this thin layer (caused by trauma or pressure from the overlying temporal lobe of the brain) may trigger both symptoms and signs. Dehiscence, being a bone defect, is nearly always diagnosed using a high-resolution CT scan on the temporal bone. Because patients show various clinical symptoms and many of them did not undergo high-resolution CT scans, SSCD is likely to be greatly underdiagnosed [2]. However, some patients also exhibit conductive hearing loss at low frequencies without vestibular symptoms. Most previous investigations have focused on clinical symptoms and treatments [3–5].

Normally, sound is transmitted through the membranes of the oval and round windows, which serve as fluid interface between air in the middle ear and the perilymphatic fluid spaces of the inner ear. The generally accepted hypothesis for the mechanism of the disease is the presence of a third window in the cochlea, which may cause energy loss during sound transmission in the inner ear. In response to the inward movement of the stapes, the membrane covering the opening is pushed outwards, which reduces the movement of the perilymph and the outward movement of the round window membrane (RWM) [6, 7].

Conductive hearing loss induced by SSCD is presumably due to an increase in air conduction thresholds, concomitant with a decrease in bone conduction thresholds. The effects of SSCD on hearing thresholds have been investigated theoretically using a lumped-element electrical circuit model [6, 8]. Recently, the relationship between the hearing threshold and the size (or location) of dehiscence has been studied to examine the mechanisms of SSCD syndrome and develop protocols for screening SSCD patients [9]. Large air-bone gaps (ABGs) have been shown to accompany SSCD at low frequencies in animal experiments (fat sand rat and chinchilla) [10, 11] and human cadaveric temporal bone studies [12].

A laser Doppler vibrometer is a noncontact, established optical technique that can be used to measure the displacement of the components of the middle ear in response to sound stimulation. This technique has been used to assess the vibration of the RWM, the tympanic membrane (TM), and the stapes footplate in fresh and embalmed cadaveric human temporal bone and animal specimens [13–15]. Over the years, animal models of different diseases have been created to investigate changes in vibration in the ossicular chain and the RWM to explore the potential mechanisms underlying the clinical symptoms of diseases.

To date, there is no *in vivo* animal model that can be used to investigate hearing loss and vibration changes with SSCD through laser Doppler velocimetry (LDV). In this study, we used the guinea pig as an experimental model, and we created dehiscence in the superior semicircular canal to simulate SSCD using a surgical technique. First, we showed that the bony fenestration of the superior semicircular canal in a guinea pig resulted in hearing loss in the low-frequency range, mimicking the characteristic hearing loss pattern in patients with SSCD. Then, we measured vibrations of the RWM, head of the stapes, umbo, and the surgically created dehiscence spot by LDV before and after fenestration, as well as after patching to test the third window hypothesis and clarify the pathogenesis of the hearing loss.

## 2. Materials and Methods

### 2.1. Animals

All animal work was approved by the institutional animal care and use committee at the Eye and Ear, Nose, and Throat Hospital, Fudan University, and complied with the National Institutes of Health guide for the care and use of laboratory animals.

In total, 36 healthy albino male guinea pigs with an initial weight of 250–300 g and a positive Preyer reflex were used. All animals were free of middle ear diseases, such as TM perforation or otitis media, as evaluated by otoscopic examination. The identification of preexisting abnormalities of auditory function was made by a prerecruiting auditory brain response (ABR) measurement for each animal. If an abnormal response was found, the animal was excluded from the study.

### 2.2. Surgical Procedure

All surgeries were performed under anesthesia by applying an anesthetic solution made by mixing 2 ml ketamine (Jiangsu Hengrui Medicine Co. Ltd., serial number 1867-66-9) with 30 mg xylazine powder (Nanjing Pharmaceutical Chemical Factory, serial number 23076-35-9), resulting in a final concentration of 15 mg/ml. The anesthetic solution was injected intramuscularly into the animal at a dose of 1 ml/kg. Supplementary doses were given as necessary, judged by the toe-pinch reflex.

**Figure 1:**
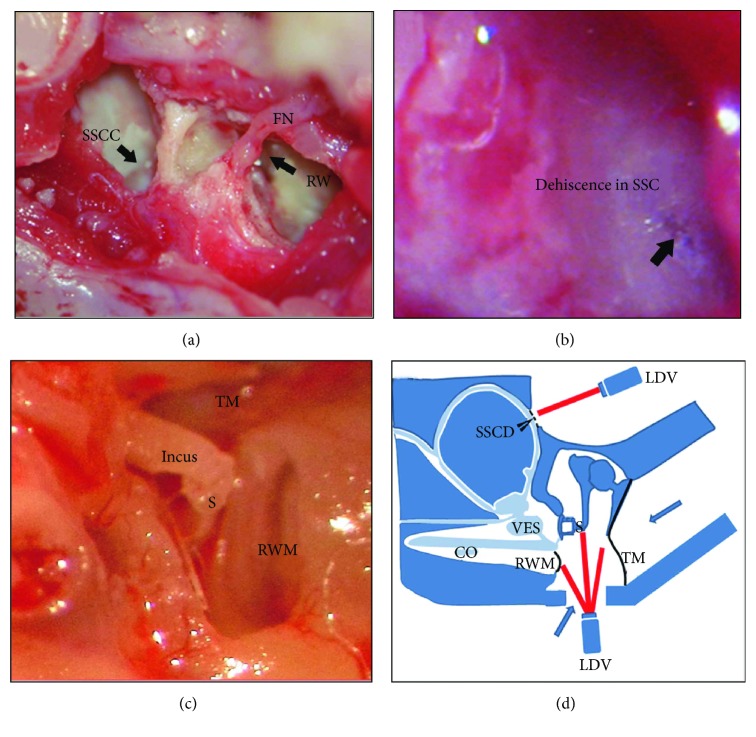
Surgical procedure for the SSCD model and schematic diagram of the laser Doppler vibrometer (LDV) detecting vibration at four locations: (a) anatomical landmarks of the superior semicircular canal (SCC), facial nerve (FN), and round window (RW), viewed from the opened middle ear cavity; (b) dehiscence created in the superior semicircular canal (SCC); (c) the incus, stapes head, round window membrane (RWM), and the medial side of the umbo, viewed from the opened middle ear cavity; (d) diagram of the LDV, as used in four locations to detect vibration.

The right ears of the experimental animals underwent surgery to simulate SSCD disorders, and the left ears served as controls. First, we surgically exposed the superior semicircular canal by opening the bulla dorsally; then, the semicircular canal bony wall was removed using a diamond burr until the membranous canals were exposed, presenting as semilucent under the microscope (Figure 1). We divided the animals based on the position of the dehiscence and its size. The individual groups were as follows: (1) a dehiscence with a size of 0.5 × 1.0 mm, close to the oval window (6 guinea pigs); (2) a dehiscence with a size of 0.5 × 0.5 mm, close to the oval window (18 guinea pigs); (3) a dehiscence with a size of 0.5 × 1.0 mm, distal to the oval window (6 guinea pigs); and (4) a dehiscence with a size of 0.5 × 0.5 mm, distal to the oval window (6 guinea pigs). We defined close to or distal to the oval window as the distances ≤ 1 mm or >3 mm from the superior semicircular canal crista, respectively, for location description.

In each animal, the vibrations of the TM (or umbo; Vu), stapes head (Vs), round window (Vw), and the place of the dehiscence (Vd) were measured (Figure 1(d)) before and after the dehiscence was created and when the dehiscence had been repaired using dental cement.

### 2.3. ABR Measurements

We have divided the individual groups as follows: (1) a dehiscence with a size of 0.5 × 1.0 mm, close to the oval window (6 guinea pigs); (2) a dehiscence with a size of 0.5 × 0.5 mm, close to the oval window (6 guinea pigs); (3) a dehiscence with a size of 0.5 × 1.0 mm, distal to the oval window (6 guinea pigs); and (4) a dehiscence with a size of 0.5 × 0.5 mm, distal to the oval window (6 guinea pigs). ABR measurement was performed before and after the dehiscence created in all four groups. ABR measurement was also performed after the dehiscence was repaired with dental cement in groups 1 and 2. After anesthesia, ABRs were tested in a soundproof booth to assess the auditory threshold (Bio-Logic NavPro, 580-NAVPR2, Natus Medical Inc., Pleasanton, CA, USA). ABR was recorded differentially using subcutaneous stainless steel electrodes placed over the rostral to the tragus of the right ear (positive), left ear (negative), and in the middle of the two ears (ground); the inter-electrode impedance was <3 kΩ. Stimulation was presented as tone bursts (5 ms duration, 0.5 ms rise/fall time, Blackman envelope) at a frequency of 0.5, 1, 2, 4, 6, and 8 kHz; the sound-intensity level was decreased in 5 dB steps from 100 to 20 dB SPL, and 1000 response traces at each sound level were recorded and averaged.

### 2.4. Laser Doppler Vibrometer Measurements

The system used in the present study included a laser Doppler vibrometer (Polytec OFV-505, Polytec, Karlsruhe, Germany) coupled with a microscope (OPMI 1-FC, Carl Zeiss, Jena, Germany), a vibrometer controller (OFV-5000, USA), a high-bandwidth backplane (PXIe-1082, NI, USA), a conditioning amplifier (MI-2004, ECON, USA), a micromanipulator (A-HLV-MM30, Polytec, Wurzburg, Germany), and a signal generator (33210A, Agilent, Santa Clara, CA, USA; Figure 1(a)). The intensity of each excitation frequency was calibrated to 100 dB SPL using a sound-level meter (AWA-5661–1B, AiHua, Yiyang city, China). An earphone associated with the microphone (ER-4PT, ER-7C, HLV-SPEC Adapter, Etymotic, Elk Grove Village, IL, USA) was inserted into the osseous external auditory canal to deliver signal stimuli and monitor sound pressure. The distance between the TM and the inserted earphone and microphone was 0.5 mm. The sound stimuli, produced by a closed loudspeaker with a frequency range of 0–10 kHz, with sound level of 100 dB SPL and duration of 320 ms (ER-4PT, USA), were delivered by a plastic tube to the TM. At the same time, a real-time sound monitor was put into the external ear canal to monitor stimulus density and sound waves. The vibration of the target surface was acquired by the system through a reflective beaded foil and recorded by a computer program (LabVIEW SignalExpress, National Instruments, USA) for further analysis. The vibration amplitude of the moving surface was calculated from the voltage output of the vibrometer's velocity decoder. Testing was conducted in a sound-treated booth to achieve a high signal-to-noise ratio. For each stimulus frequency, sound stimuli were repeated at least three times. Traces presenting a good signal-to-noise ratio were averaged during offline data analysis.

### 2.5. Section Processing and Hematoxylin and Eosin Staining

Six guinea pigs were used in this part. The right ears of three animals underwent successful surgery to simulate SSCD disorders with a thin endosteum in the dehiscence. Surgeries on the other three animals failed resulting in fistula in SSCD. Intracardiac perfusion was performed with 150 ml 0.2 M PBS, followed by 4% paraformaldehyde, and then the temporal bone was removed and fixed in 4% paraformaldehyde (pH = 7.4) for more than 24 h at 4°C. The temporal bones were decalcified in ethylenediaminetetraacetic acid. Samples were saturated in 15% sucrose for 4 h and then 30% for 4 h, embedded in optimum cutting temperature (OCT) compound, and sectioned serially at 10 *μ*m in the plane perpendicular to the SCD (Leica CM1830). The sections were stained with hematoxylin and eosin (H&E) and then were observed under a light microscope (6030116204, Carl Zeiss, Jena, Germany).

### 2.6. Statistical Analysis

Data are presented as mean ± standard error. All analyses were performed using SPSS software (ver. 19.0; IBM, USA). Two-tailed Student's *t*-tests were used to determine the confidence interval for comparison between two groups. *P* values < 0.05 were considered statistical significance.

## 3. Results

### 3.1. Observation of Tissue Sections of Surgically Induced SSCD in Guinea Pigs

**Figure 2:**
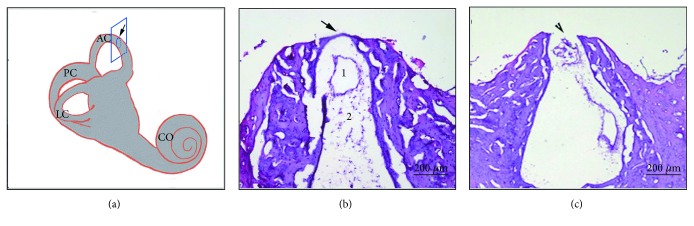
Successful SSCD model, confirmed by sectioning and hematoxylin and eosin (H&E) staining. (a) shows the schematic diagram of the section plane (blue pane) of the fenestration (arrow) in SCD. The section shows a successful SSCD model in the plane perpendicular to the superior semicircular canal dehiscence with H&E staining showing the thin endosteum in the dehiscence ((a) 1 = endolymph fluid space, 2 = perilymph fluid space) and a failed SSCD model with a large fistula (b).

To understand the successful surgeries that created dehiscence in the superior semicircular canal, we observed the sections after H&E staining. In the right ear of the animal, we created dehiscence close (1 mm) to the oval window with a size of 0.5 × 0.5 mm (Figure 1(b)). Under the light microscope, we observed that the membranous canal was exposed and appeared semilucent (Figure 1(b)). The criterion for success in the dehiscence model was a thin endosteum in the dehiscence (seen in the schematic diagram of the section plane (Figure 2(a)) and H&E staining; Figure 2(b)), demonstrating clearly the fluid areas of the endolymph and perilymph. A surgeon should refrain from removing too much bone from the superior semicircular canal, which could result in a large fistula (e.g., Figure 2(c)) and false measurement of lymph pressure. In the supplemental material, we have done the whole-mount immunostaining with phalloidin (green), peripherin (red), and Ctbp2 (blue) in the apical (S1: A, D, and G), middle (S1: B, E, and H), and midbasal (S1: C, F, and I) turns of the cochlear in the surgically prepared guinea pigs (dehiscence close to the oval window with a size of 0.5 × 0.5 mm), which showed the normal structure of hair cells, neural nerves, and synapses.

### 3.2. Effects of SSCD and Its Repair on ABR Response

In this experiment, the animals underwent surgeries to create dehiscence close to the oval window and distal to the oval window, with a size of 1.0 × 0.5 mm and 0.5 × 0.5 mm, within 1 mm or more than 3 mm from the superior semicircular canal crista. ABR threshold measurements were performed before and after the dehiscence was created in the four groups; ABR measurement was also performed after the dehiscence was repaired with dental cement with the dehiscence close to the oval window.

**Figure 3:**
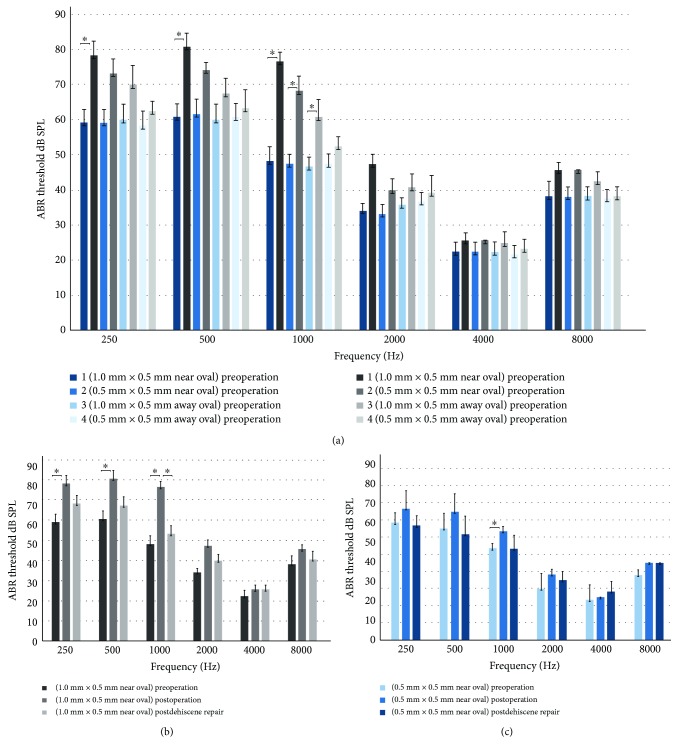
ABR threshold in different groups at frequencies of 0.25, 0.5, 1, 2, 4, and 8 kHz. (a) shows the ABR thresholds in four groups (1: 1 × 0.5 mm near the oval (*n* = 6), 2: 0.5 × 0.5 mm near the oval (*n* = 6), 3: 1 × 0.5 mm far away from the oval (*n* = 6), and 4: 0.5 × 0.5 mm far away from the oval (*n* = 6)) at frequencies of 0.25, 0.5, 1, 2, 4, and 8 kHz. ABR threshold in all frequencies increased after the dehiscence was created in group 1 and group 2, with a statistically significant difference at the frequency of 250, 500, and 1000 Hz (^∗^ *P* < 0.05) in group 1 (^∗^ *P* < 0.05) and 1000 Hz in group 2 (^∗^ *P* < 0.05). (b) and (c) show ABR threshold presurgery, postsurgery SSCD, and postdehiscence repair at frequencies of 0.25, 0.5, 1, 2, 4, and 8 kHz in groups 1 and 2. The ABR thresholds at the low frequencies 250, 500, 1000, and 2000 Hz increased notably after the dehiscence (1 × 0.5 mm near the oval and 0.5 × 0.5 mm near the oval) was created, with a statistically significant difference at the frequency of 250, 500, and 1000 Hz in group1 (^∗^ *P* < 0.05) and 1000 Hz in group 2 (^∗^ *P* < 0.05), which was offset after the dehiscence was repaired.

The results of the ABR threshold before the operation, in the SSCD model, and after patching the dehiscence are listed in Figure 3. The means and standard errors of all groups were recorded. The elevation of the ABR threshold was observed in ears associated with dehiscence in the superior semicircular canal in the four groups (*n* = 6 in each group) at low frequencies of 250, 500, 1000, and 2000 Hz (Figure 3(a)). Student's *t*-tests showed that at 250, 500, and 1000 Hz, the mean ABR threshold in the SSCD model (0.5 × 1.0 mm, close to the oval window) was significantly higher than it was before the operation (*P* < 0.05, indicated by the asterisks in Figures 3(a) and 3(b)). In the SSCD model (0.5 × 0.5 mm close to the oval window), the mean ABR threshold in 1000 Hz was significantly higher than it was before the operation. (*P* < 0.05, indicated by the asterisks in Figures 3(a) and 3(c)). There were no significant ABR threshold elevation in the dehiscence far away from the oval window. After the dehiscence close to the oval window was repaired, the ABR thresholds were decreased at frequencies of 250, 500, 1000, and 2000 Hz (Figures 3(b) and 3(c)). Student's *t*-tests revealed that the mean ABR threshold in the dehiscence close to the oval window after patching showed no difference compared to the group before the operation (*P* > 0.05).

### 3.3. Effects of SSCD and Its Repair on Movement of the Umbo, Stapes Head, and RWM and the Effects of Location of Dehiscence according to LDV Measurements

In this experiment, the animals used were the same as those for the ABR threshold measurements, with dehiscence close to the oval window, with a size of 0.5 × 0.5 mm (*n* = 6). After ABR threshold measurement, the animals underwent LDV measurement.

A laser vibrometer was used to measure the target vibration. Vu, Vs, Vw, and Vd were detected (Figures 1(c) and 1(d)). During the measurements, a foil with reflective beads was attached to the target position to gain robust signals. To determine the SSCD influence, we measured and compared vibrations at the four different locations before and after the dehiscence was created as well as after the dehiscence was repaired with dental cement.

**Figure 4:**
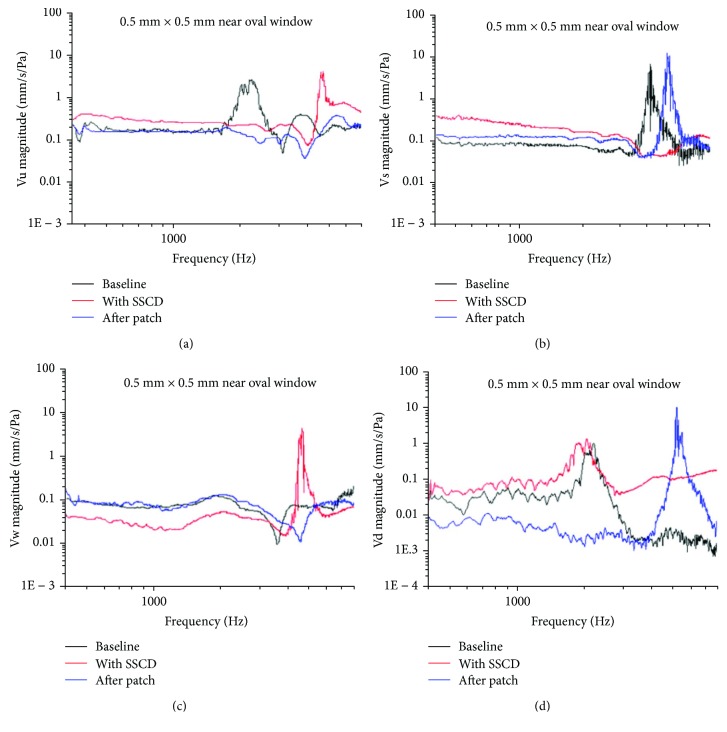
Vibration of the tympanic membrane (a), stapes head (b), round window (c), and the dehiscence (d), detected by LDV presurgery (black baseline) and postsurgery (red line) and after the dehiscence was repaired (blue line) (*n* = 6). (a) The presence of dehiscence (red line) statistically significantly (*P* < 0.006) increased the vibration of the tympanic membrane, in the range of 300–1500 Hz. The increase showed an offset after the dehiscence was repaired after surgery (blue line). (b) The presence of the dehiscence (red line) increased the vibration of the staples head, whereas it decreased the inner ear impedance statistically significantly (*P* < 0.001) in the range of 300–3000 Hz. This increase showed an offset after the dehiscence was repaired. (c) The presence of the dehiscence (red line) decreased vibration in the round window membrane statistically significantly (*P* < 0.001), in the range of 300–3000 Hz, which was offset after the dehiscence was repaired (blue line). (d) The vibration of the dehiscence located at the superior semicircular canal increased statistically significantly (*P* < 0.001) after the dehiscence was created, in the range of 300–2000 Hz. This increase showed an offset after the dehiscence was repaired.

There was some energy leakage from the created third window, in the range of frequencies from 300 to 1500 Hz in the animal model, which presented as increased Vu (Figure 4(a)) and Vs (Figure 4(b)). In Figure 4(a), it can be seen that in comparison with the baseline (black line), the presence of dehiscence (red line) resulted in increased Vu significantly (*P* < 0.006), in the frequency range of 300–1500 Hz. The increase showed an offset after the dehiscence was repaired after surgery (blue line). In Figure 4(b), the presence of the dehiscence resulted in increased Vs, whereas it led to significantly decreased inner ear impedance (*P* < 0.001), in the range of 300–3000 Hz. This increase showed an offset after the dehiscence was repaired.

The decreased Vw (Figure 4(c)) resulted in statistically significantly decreased (*P* < 0.001) inner ear impedance, in the range of 300–3000 Hz. The decrease showed an offset after patching.

Vd, located at the superior semicircular canal (Figure 4(d)), increased significantly (*P* < 0.001) after the dehiscence was created, in the range of 300–2000 Hz. This increase showed an offset after the dehiscence was repaired. The energy leakage was improved after the dehiscence was repaired.

### 3.4. Effects of SSCD with Varied Sizes and Locations on Movement of the Umbo, Stapes Head, and RWM, Assessed by LDV Measurements

In this part, the two dehiscence sizes were 0.5 × 0.5 mm and 0.5 × 1.0 mm, while the two locations were near the oval window (less than 1 mm between the superior semicircular canal crista) and away from it (3 mm from the superior semicircular canal crista). Vu, Vs, and Vw were measured before and after the dehiscence were created (*n* = 6 in each group).

**Figure 5:**
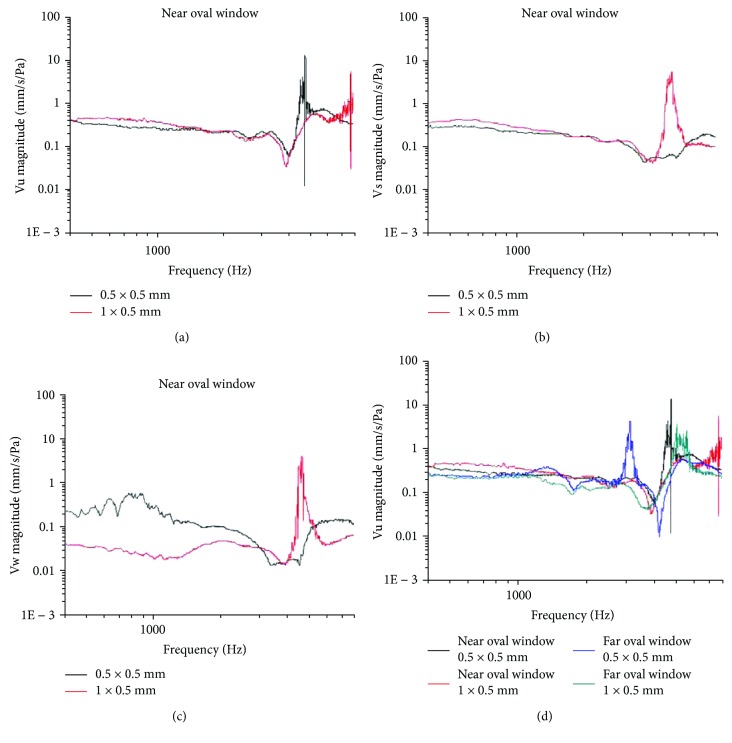
Effects of SSCD with different sizes and locations on the movement of the tympanic membrane, stapes head, and RWM by LDV measurements presurgery and postsurgery SSCD. (a) Vibration of the tympanic membrane (Vu) in the groups with different sizes of dehiscence (0.5 × 0.5 mm and 1.0 × 0.5 mm) near the oval window. Vu in the group with the larger dehiscence (1.0 × 0.5 mm; red line) was stronger than in the group with the smaller dehiscence (0.5 × 0.5 mm; black line), in the range of 500–1000 Hz (*P* < 0.05). (b) Vibration of the stapes head (Vs) in groups with different sizes of dehiscence (0.5 × 0.5 mm and 1.0 × 0.5 mm) near the oval window. Vs in the group with the larger dehiscence (1.0 × 0.5 mm; red line) was stronger than in the group with the smaller dehiscence (0.5 × 0.5 mm; black line), in the range of 400–1000 Hz (*P* < 0.05). (c) Vibration of the round window (Vw) in groups with different sizes of dehiscence (0.5 × 0.5 mm and 1.0 × 0.5 mm) near the oval window. Vw in the group with the larger dehiscence (1.0 × 0.5 mm; red line) was weaker than in the group with the smaller dehiscence (0.5 × 0.5 mm; black line), in the range of 400–2000 Hz (*P* < 0.001). (d) Vibration of the tympanic membrane in the groups with different sizes of dehiscence located at different points (*n* = 6 in each group). Vibration in the group with the larger dehiscence (1.0 × 0.5 mm; red line) was stronger than in the group with the smaller dehiscence (0.5 × 0.5 mm; black line) near the oval window (*P* < 0.05) in the range of 500–1000 Hz. Vibration in the group with the smaller dehiscence (0.5 × 0.5 mm; black line) near the oval window was stronger than in the groups with larger (green line) or small dehiscences (blue line) far from the oval window (*P* < 0.05) in the range of 400–800 Hz. There were no statistically significant differences between the groups with different sized dehiscences away from the oval window (*P* > 0.05).

Vu in the group with the larger dehiscence (1.0 × 0.5 mm; red line) was stronger than in the group with the smaller dehiscence (0.5 × 0.5 mm; black line), in the range of 500–1000 Hz (*P* < 0.05; Figure 5(a)). Vs in the group with the larger dehiscence (1.0 × 0.5 mm; red line) was stronger than in the group with the smaller dehiscence (0.5 × 0.5 mm; black line), in the range of 400–1000 Hz (*P* < 0.05; Figure 5(b)). Vw in the group with the larger dehiscence (1.0 × 0.5 mm; red line) was weaker than in the group with the smaller dehiscence (0.5 × 0.5 mm; black line), in the range of 400–2000 Hz (*P* < 0.001; Figure 5(c)).

Vu in the group with the large dehiscence (1.0 × 0.5 mm; red line in Figure 5(d)) near the oval window was stronger than in the group with the smaller dehiscence (0.5 × 0.5 mm; black line in Figure 5(d)) near the oval window, in the range of 500–1000 Hz (*P* < 0.05). Vu in the group with the smaller dehiscence (0.5 × 0.5 mm; black line in Figure 5(d)) near the oval window was stronger than in the group with the smaller dehiscence (0.5 × 0.5 mm; blue line in Figure 5(d)) and the larger dehiscence (1.0 × 0.5 mm; green line in Figure 5(d)) far from the oval window, in the range of 400–800 Hz (*P* < 0.05). There were no statistically significant differences (*P* > 0.05) between the groups with different size dehiscences located far from the oval window (blue and green lines in Figure 5(d)).

Generally, the impedance of the inner ear in the group with a dehiscence of 1.0 × 0.5 mm located close to the oval window had larger decreases than the group with a dehiscence of 0.5 × 0.5 mm located at the same site in the frequency range of 500–1000 Hz. The vibration coming from the group with a dehiscence close to the oval window decreased more than the others in the frequency range of 400–800 Hz.

## 4. Discussion

This is, to the best of our knowledge, the first report on the measurement of movements of the umbo, stapes head, and RWM *in vivo* in a guinea pig SSCD model using an LDV. The LDV has become a popular device for measuring sound-induced TM velocity in healthy ears and ears with conductive hearing loss for diagnostic purposes in the clinic [16, 17]. In experimental settings, LDV is also used to measure other membranous movements. For example, the vibration of the RWM associated with acute otitis media is significantly decreased compared to that in the healthy ears of guinea pigs [15]. However, no prior study has reported dynamic behavioral changes in the umbo, the stapes head, and the RWM in association with the SSCD animal model *in vivo*.

Previous studies have suggested a third window hypothesis for the SSCD syndrome. Briefly, the vibration of the stapes footplate would induce motion of the endolymph in the bony semicircular canal when a third window exists [6, 18]. Although the third-window hypothesis is useful for explaining our experimental results and many symptoms observed in patients with SSCD syndrome, it is unclear how it resolves the vast variability in symptoms in SSCD patients mechanistically. Some SSCD patients show debilitating vestibular symptoms, such as a severe sound-induced or pressure-induced vertigo with normal hearing; others have hearing loss, such as conductive, sensorineural, or mixed, with no significant vestibular symptoms; and some patients seem to have a combination of vestibular and auditory symptoms [3, 6–8, 19, 20]. What is the mechanistic basis for these different clinical symptoms? Are the varying sizes and locations of the dehiscences in SCD responsible for the different hearing and vestibular symptoms? A clinical study found no significant association between the size or location of the dehiscence and the audiogram pattern or individual findings in 27 patients (34 ears) [21]. However, Yuen et al. reported that an ABG was seen consistently at low frequencies when the dehiscence was larger than 3 mm and that the size of the average ABG correlated with the size of the dehiscence [22]. Sone et al. evaluated five ears with pathological third-window lesions. Contrast-enhanced magnetic resonance imaging (MRI) revealed that four had endolymphatic hydrops (EH), which might create auditory or vestibular symptoms [23].

To clarify this phenomenon, dehiscences of various sizes and locations along the superior semicircular canal were made in guinea pigs. Then, at various locations in the ear, we measured the vibration resulting from application stimuli of various frequencies using LDV. In our experiment, we found that animals with larger dehiscences (0.5 × 1.0 mm) showed larger effects on inner ear impedance than those with smaller dehiscences (0.5 × 0.5 mm). In investigating the effects of dehiscence location on the symptoms, we found that a dehiscence close to the oval window produced more effects on the inner ear system than a dehiscence that was further away. This result is similar to that of a previous three-dimensional finite-element model of a human ear study, which showed the importance of the width of the dehiscence closest to the oval window [24]. In clinical SSCD patients, various circumstances affect hearing and vestibular symptoms, not only the size and location of the dehiscence. Furthermore, in our guinea pig SSCD model, there was some energy leakage from the third window created, in the range of frequencies of 300–1500 Hz, which presented as increased Vu, Vs, and Vd, as well as decreased Vw, resulting in a decreased inner ear impedance in the range of 300–3000 Hz, consistent with the hypothesis that a dehiscence acts as a mobile third window, in addition to the oval and round windows, providing a pathway through which a fluid-motion wave can be shunted away from the cochlea into the SSC. After patching the fenestration, the increased Vu, Vs, and Vd showed offsets, and simultaneously, Vw increased back to almost normal. One limitation of our study is that we did not measure changes in vestibular function in this model with fenestration and after patching the fenestration. Our aim is to further explore the hearing loss mechanism *in vivo* in this animal model and determine whether it is related to the decrease in inner ear impedance. Furthermore, it remains difficult to evaluate and quantify vestibular symptoms fully using present devices with this animal model.

According to clinical auditory symptoms, many studies have concluded that typical patients demonstrate low-frequency conductive hearing loss, which can be rectified after surgery [3, 9, 17, 25–27]. Our *in vivo* SSCD model demonstrated the same phenomenon. In our experiment, we also found that the ABR thresholds increased after the dehiscence was created at low frequencies, namely, 250, 500, 1000, and 2000 Hz, and particularly at 250, 500, and 1000 Hz with the dehiscence 1.0 × 0.5 mm and at 1000 Hz with the dehiscence 1.0 × 0.5 mm close to the oval window (significant increase). These ABR threshold increases all showed offsets after the fenestrations close to the oval window were repaired surgically. This could be explained by the presence of a third window with decreased cochlear impedance due to energy leakage. After the fenestration of the SSC was patched, the third window was eliminated and the fluid motion in the SSC reverted to normal.

In conclusion, the presence of SSCD, behaving as a third window in the inner ear, could cause energy leakage during sound transmission, thus lowering inner ear impedance, resulting in corresponding cochlear symptoms. The size and location of the dehiscence appear to be important in producing differential effects in the pathology.

## Supplementary Materials

Supplementary Materials S1: whole-mount immunostaining of cochlear basement membrane in the surgically prepared guinea pigs (dehiscence close to the oval window with a size of 0.5 × 0.5 mm), with phalloidin (green), peripherin (red), and Ctbp2 (blue) in the apical (A, D, and G), middle (B, E, and H), and midbasal (C, F, and I) turns of the cochlear, which showed the normal structure of hair cells, neural nerves, and synapses.
